# Regulation of microRNAs miR-30a and miR-143 in cerebral vasculature after experimental subarachnoid hemorrhage in rats

**DOI:** 10.1186/s12864-015-1341-7

**Published:** 2015-02-22

**Authors:** Anne Holt Müller, Gro Klitgaard Povlsen, Claus Heiner Bang-Berthelsen, Lars Schack Kruse, Janne Nielsen, Karin Warfvinge, Lars Edvinsson

**Affiliations:** Department of Clinical Experimental Research, Glostrup Research Institute, Glostrup University Hospital, Nordre Ringvej 69, Glostrup, 2600 Denmark

**Keywords:** SAH, Animal model, Artery, Biomarker, Non-coding RNA

## Abstract

**Background:**

microRNAs (miRNAs) are important regulators of translation and have been implicated in the pathogenesis of a number of cardiovascular diseases, including stroke, and suggested as possible prognostic biomarkers. Our aim was to identify miRNAs that are differentially regulated in cerebral arteries after subarachnoid hemorrhage (SAH), using a rat injection model of SAH and a qPCR-based screen of 728 rat miRNAs. Additionally, serum was analyzed for a possible spill-over to the circulation of regulated miRNAs from the vessel walls.

**Results:**

We identified 482 different miRNAs expressed in cerebral arteries post-SAH. Two miRNAs, miR-30a and miR-143, were significantly upregulated in cerebral arteries after SAH when compared to sham-operated animals. However, none of these exhibited significantly altered serum levels after SAH versus post-sham surgery. The most robust upregulation was seen for miR-143, which has several predicted targets and is a strong regulator of vascular morphology. We hypothesize that miR-30a and miR-143 may play a role in the vascular wall changes seen after SAH.

**Conclusions:**

We report that miR-30a and miR-143 in the cerebral arteries show significant changes over time after SAH, but do not differ from sham-operated rats at 24 h post-SAH. Although this finding suggests interesting novel possible mechanisms involved in post-SAH cerebrovascular changes, the lack of regulation of these miRNAs in serum excludes their use as blood-borne biomarkers for cerebrovascular changes following SAH.

**Electronic supplementary material:**

The online version of this article (doi:10.1186/s12864-015-1341-7) contains supplementary material, which is available to authorized users.

## Background

Stroke is a leading cause of death and disability worldwide, and stroke patients have a case fatality risk that is approximately three times higher than that of patients who suffer a coronary infarct. Subarachnoid hemorrhage (SAH) is a type of stroke associated with vasospasm, late cerebral ischemia (LCI), and a high death rate [1]. We have found that LCI correlates with upregulation of contractile receptors and inflammation in brain vessels and that these changes rely on enhanced transcription and translation of specific genes in the vascular smooth muscle cells of cerebral arteries [2].

Non-coding RNAs comprise multiple classes of RNA transcripts that are not translated into proteins but instead regulate the stability and translation of protein-coding transcripts. The most studied of these are the microRNAs (miRNAs), consisting of 19–24 nucleotides. miRNAs modulate gene expression through both mRNA degradation and translational repression mechanisms, and miRNA–mRNA regulatory networks are highly complex [3]. Mature miRNAs are incorporated into the RNA-induced silencing complex (RISC), where they can bind to their target mRNA [4]. This binding may result in inhibition of translation or deadenylation of the mRNA and thus promotion of degradation [5]. Studies have also suggested that miRNAs can target promoter regions and induce gene expression [6]. Each miRNA might have hundreds of targets and the potential to regulate many biological processes such as apoptosis [7] and the immune response [8]. miRNAs are expressed at different levels in different cell types, and these levels change under pathological conditions [9].

miRNAs also play an important role in regulating vascular smooth muscle phenotype by maintaining or inhibiting differentiation [10]. Furthermore, miRNAs are thought to be involved in the pathophysiology of different forms of stroke. In rats, blood and cortical miRNA levels change dramatically after experimental focal ischemic stroke [11] and after intracerebral hemorrhage [12]. In ischemic stroke patients, the levels of certain miRNAs in the blood are associated with clinical outcome [13]. Some of the highly expressed miRNAs in post-stroke brain tissue can be detected in the peripheral blood [12,14], indicating that brain-specific miRNAs could serve as blood-borne biomarkers for brain ischemia [15]. In the case of SAH, there is an unmet need for blood-borne biomarkers for predicting risk of cerebral vasospasm and LCI.

A series of earlier studies demonstrated a number of expressional changes in cerebral arteries after different types of stroke, such as focal cerebral ischemia [16] and SAH [17], and after cardiac arrest [18]. We have shown that activation of specific signal transduction pathways in the cerebral vasculature after stroke leads to transcriptional regulation of vasoconstrictor receptors, inflammatory mediators, and proteins involved in maintaining blood–brain barrier integrity [19]. However, regulation likely also occurs at other levels, and we hypothesize that SAH leads to changes in miRNAs in cerebral arteries and that these miRNAs are secreted into serum where they may serve as biomarkers for predicting risk of cerebral vasospasm and LCI.

Thus, the aim of this study was to examine and characterize changes in miRNA levels in cerebral vessels from rats after experimental SAH as compared to sham-operated animals, and to establish if altered miRNA levels are detectable in serum. We report that miR-30a, involved in regulation of angiogenesis, and miR-143, correlated to vascular smooth muscle tone regulation, are significantly regulated after SAH.

## Results

### Experimental SAH

For the miRNAome screening, three groups of animals were subjected to experimental SAH and terminated at different time points (1 h, 6 h, and 24 h) after surgery. A fourth group of animals was subjected to sham operation, and terminated at 24 h post-surgery, and used as controls. For the technical confirmation part of the study and for serum investigations, a fifth group of animals, termed 0 h, was also included; these animals were terminated immediately after injection of blood. Each group contained four replicates, and each replicate consisted of tissue pooled from two animals; i.e., eight animals were used for each group. During SAH surgery, intracranial pressure (ICP) went from 4–8 mmHg to 80–100 mmHg following injection of blood. The cortical blood flow was reduced by a minimum of 75% and remained low for a minimum of 5 min after blood injection. In addition, a Cushing effect was observed in all animals. These physiological parameters of experimental SAH reached values similar to those we have previously described [20] and did not differ between the groups.

### miRNAome screening of large cerebral arteries

**Figure 1:**
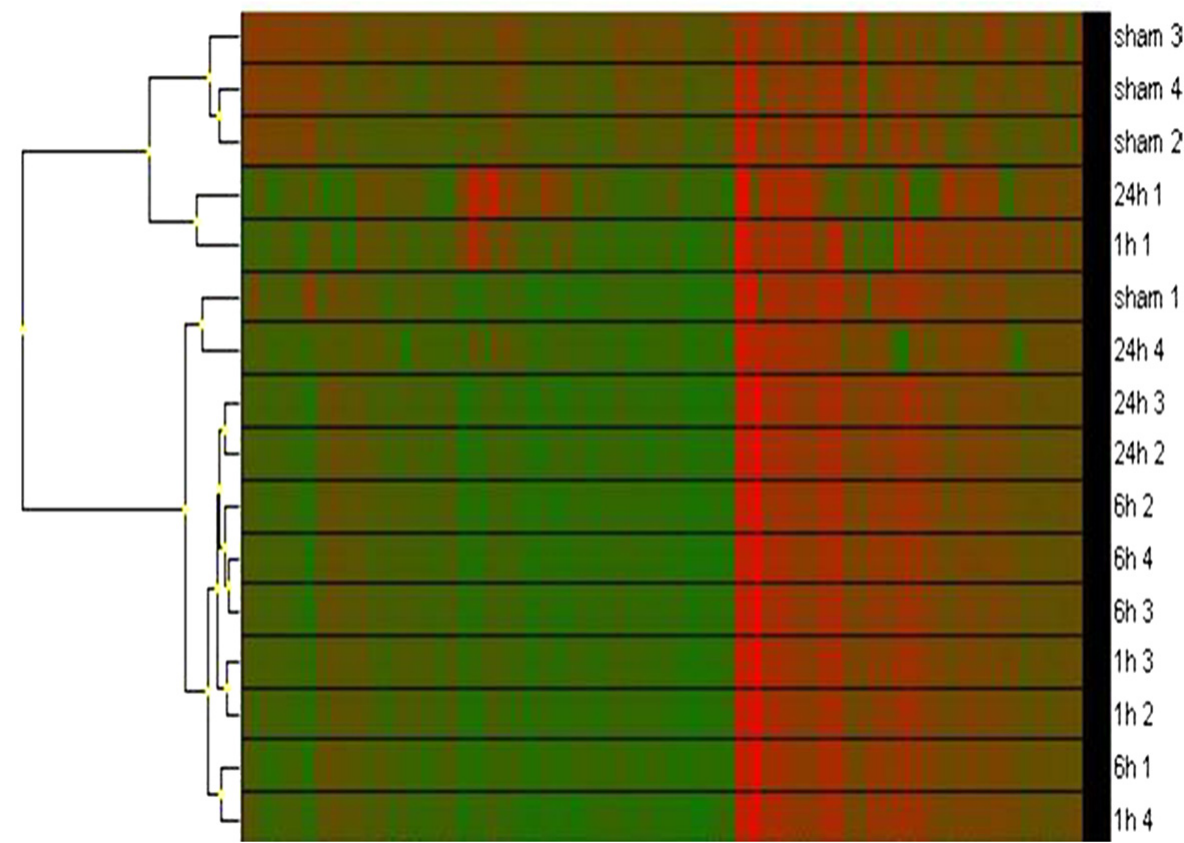
**Cerebral arterial miRNAs expressed after SAH.** Hierarchical cluster analysis of miRNAs expressed in large cerebral arteries of rats subjected to SAH for 1 h, 6 h, or 24 h or to sham surgery. The later time points (24 h post-SAH or post-sham) cluster together and away from the early time points (1 h and 6 h post-SAH). Red indicates high expression and green low expression.

The initial screen of 728 rat miRNAs from cerebral vessels demonstrated that 482 miRNAs were expressed in both sham and SAH rats at 1, 6, and 24 h post-operation (Additional file 1). The overall analysis of the data from the initial screen revealed differences in miRNA expression between sham-operated animals and animals at the early stages of SAH (1 h and 6 h post-SAH). Hierarchical clustering analysis showed that samples from sham-operated animals and 24-h SAH animals clustered together and that samples from 1-h and 6-h SAH animals clustered away from samples from the late time point (24 h post-SAH) (Figure 1). Of the 482 miRNAs that were expressed in both sham-operated animals and animals subjected to SAH, we found that 4 miRNAs (miR-30a, miR-143, miR-191*, and miR-223) showed statistically significant changes in expression between the experimental groups (Table 1).

**Table 1:** **Cerebral arterial miRNAs significantly changed after SAH**

	**1 h**	**6 h**	**24 h**	**ANOVA p-value**
**miR-30a**	0.5 ± 0.07	2.0 ± 0.5	0.6 ± 0.05	0.005
**miR-143**	0.1 ± 0.01	16.3 ± 4.8	0.7 ± 0.5	0.001
miR-191*	6.3 ± 2.0	10.2 ± 3.0	6.5 ± 1.8	0.04
**miR-223**	5.4 ± 1.3	0.7 ± 0.05	1.7 ± 0.9	0.02

Fold changes over sham in large cerebral arteries for miR-30a, miR-143, miR-191*, and miR-223 at 1 h, 6 h, and 24 h post-SAH. Data from the miRNAome screen were analyzed using the 2^–ΔCt^ quantitative method and one-way ANOVA. Of the 482 miRNAs expressed in both SAH and sham animals, the 4 miRNAs listed in this table showed significantly different expression levels between the groups. The p values from the ANOVA analyses are in the right column.

### Confirmation of regulation of miRNA levels in cerebral arteries

**Figure 2:**
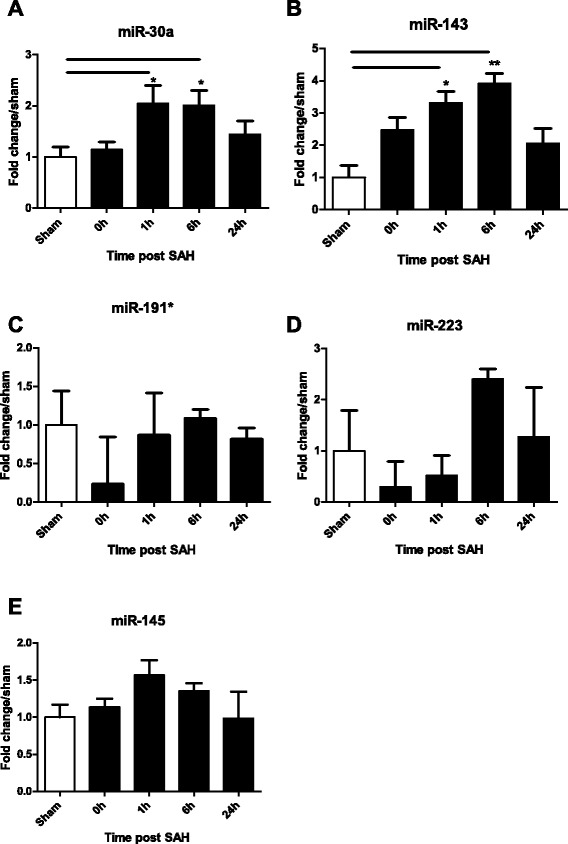
**Confirmation of differential miRNA expression.** To confirm the differential expression of miR-30a, miR-143, miR-191*, and miR-223 in SAH and sham animals as well as the lack of differential expression of miR-145, additional qPCR assays were performed. Fold changes over sham were calculated using the 2^–ΔΔCt^ quantitative method and analyzed with Student’s t-tests. The differential expression of miR-191* and miR-223 could not be confirmed. Fold change over sham for miR-30a **(A)**, miR-143 **(B)**, miR-191* **(C)**, miR-223 **(D)**, and miR-145 **(E)** at 0 h, 1 h, 6 h, and 24 h post-SAH. Sham (white bars) is set to 1. * = p < 0.05, ** = p < 0.01, compared to sham.

Subsequent technical confirmation of the data with additional qPCR assays confirmed that miR-30a and miR-143 exhibited significantly altered expression levels after SAH when compared to sham animals (Figure 2). miR-30a and miR-143 were both significantly upregulated at 1 h and 6 h post-SAH as compared to sham. The increases were approximately 2-fold for miR-30a at both time points and 3- and 4-fold, respectively, for miR-143. For both miR-30a and miR-143, the SAH-induced upregulation appeared to be transient because expression levels at 24 h post-SAH did not differ from sham. The other two miRNAs identified in the miRNA screen, miR-191* and miR-223, showed no significant differences in expression between the sham and the other groups (Figure 2).

We also examined the regulation of miR-145 because of its relationship with miR-143 and because many studies have shown that the two often co-transcribe and are important in the phenotype of vascular smooth muscle cells [10,21]. However, we could not demonstrate any regulation of miR-145 in this study (Figure 2E).

### miRNA expression in serum after SAH

As mentioned previously, miRNAs have been suggested as potential disease biomarkers because they can be detected in peripheral blood [13]. To examine the potential use of miRNAs as biomarkers for cerebrovascular pathology after SAH, possible spill-over of regulated miRNAs from cerebral arteries into the bloodstream was investigated. Total blood was obtained from rats subjected to experimental SAH and sham surgery, and the miRNA levels of 12 selected candidate miRNAs including miR-30a, miR-143, and miR-223 were measured along with 9 controls for normalization (Table 2). The candidates were selected based on the initial screen of miRNA expression in cerebral arteries and on previous findings. miR-145, miR-221, and miR-222 were selected based on their relationship with miR-143 and miR-223, respectively. miR-133a was chosen because of its known involvement in controlling the phenotypic switch in vascular smooth muscle cells [22,23]. miR-21, a marker of vascular smooth muscle cell proliferation and apoptosis, was also included along with miR-126, an endothelial marker, and miR-320, which is linked to ischemia and infarction [24-26].

**Table 2:** **miRNAs investigated in serum**

**Candidates**	**Normalizers**
*Rno-miR-143-3p*	Rno-miR-23a-3p
Rno-miR-145-5p	Rno-miR-24-5p
Rno-miR-221-3p	Rno-miR-24-3p
Rno-miR-222-3p	Rno-miR-181a-1-3p
*Rno-miR-223-3p*	Rno-miR-181a-5p
*Rno-miR-30c-5p*	Rno-miR-103-3p
Rno-miR-133a-5p	Rno-miR-107-3p
Rno-miR-133a-3p	Rno-let-7c
Rno-miR-21-5p	Rno-miR-451a-5p
Rno-miR-126a-3p	
Rno-miR-126a-5p	
Rno-miR-320a-3p	

Serum was investigated for the potential spill-over of miRNA from the cerebral arteries. The table shows selected candidate miRNAs (left column) and normalizer miRNAs (right column). Candidate miRNAs identified in the initial screen of miRNA expression in cerebral vessels are shown in italics.

Because the 0 h and 1 h post-SAH time points are of little relevance in a biomarker setting, we focused on miRNA levels in serum at 6 h and 24 h post-SAH, when a biomarker would be therapeutically relevant. None of the investigated miRNAs showed significant changes in serum levels at these time points when compared to sham (data not shown), and the increased levels of miR-30a and miR-143 found in the arteries post-SAH were thus not reflected in serum levels.

## Discussion

The present study is the first demonstration of a time-dependent change in the expression of miR-30a and miR-143 in large cerebral arteries after experimental SAH in rats. This finding adds important information to our current knowledge of the vascular events that occur in cerebral arteries following acute SAH. We used a screening method to assess the whole rat miRNAome to identify as many miRNAs as possible in an un-biased setting (n = 728). This choice made it possible to identify four miRNAs that were differentially expressed in cerebral arteries between the experimental groups. Subsequent additional qPCR assays confirmed the changes identified for miR-30a and miR-143, but not for miR-191* and miR-223.

To investigate the time-course of changes in cerebrovascular miRNA expression after SAH, we conducted the screen at three time points post-SAH and compared the results to those for sham-operated animals. In the current study only one group of sham-operated animals were included, i.e. rats that were terminated 24 h after sham surgery. It could be argued that sham groups should have been included for each time point in order to strengthen the conclusion that the here presented miRNA changes are indeed due to SAH and not surgery per se. However, we believe that it is fair to assume that for the applied SAH model the sham surgery does not induce major molecular changes in the animals. This assumption is based on our previous research [27] where there are no significant differences in mRNA levels of IL6, STAT3 and JAK2 between animals subjected to sham surgery for the SAH model used in our study and terminated 6 h and 24 h after surgery. Additionally, levels of phosphorylated STAT in sham-operated animals are equivalent in 6-h sham and 24-h sham animals [28] and detailed analyses of mRNA changes of the three housekeeping genes GAPDH, β-actin and EF-1 (elongating factor 1) in cerebral arteries after SAH [29-31] demonstrated the same stable expression at all time points studied (0, 1, 3, 6, 12, 24 and 48 hours).

At 6 h post-SAH, the miRNA changes were most pronounced. At 24 h after SAH, miRNA levels had returned to baseline and did not differ from values for corresponding miRNA in sham-operated animals. miR-30a and miR-143 both displayed upregulation in cerebral arteries at 6 h after SAH. The role of the upregulation or downregulation of a given miRNA depends on whether it is positively or negatively regulating protein expression at the post-translational level by binding to the 3’untranslated region of its target mRNA [3] or acts by another mechanism, such as inducing expression by binding to specific promoter regions in the nucleus [6].

The finding presented here is most intriguing because miR-143 is highly expressed in vascular smooth muscle cells, cardiac muscle, and endothelial cells and has been identified as essential for regulating smooth muscle cell proliferation and differentiation [32]. We have not directly tested whether the observed changes in miR-143 expression after SAH are pathological or beneficial. However, the fact that miR-143 was identified in our screen strengthens the confidence in this miRNA as an important contributor to the vascular changes that occur in conjunction with experimental SAH. Interestingly, miR143/145 knockout mice are viable and fertile, but they have a thinner arterial wall than the wild-type animals and thus significantly lower blood pressure [33]. Furthermore, neointima formation in response to vascular injury is profoundly impeded in mice lacking these miRNAs [34]. In a conditional Dicer knockout mouse, which do not have functional miRNAs, it was found that miRNAs are essential for vascular smooth muscle cell differentiation and function, a phenotype that can partially be rescued by miR-145, again pointing to its crucial role in vascular smooth muscle cells [32,35].

A hallmark and a concerning complication of SAH is excessive cerebral vasoconstriction in the hours and days after insult, with the development of LCI [36]. This study suggests that an increase in miR-143 and in part miR-145 in the hours after SAH could play an important role in the early development of this pathology. However, this possibility needs to be investigated further in studies in which, for instance, the effect of miR-143 antagomirs on vasoconstriction after SAH could be evaluated.

The altered regulation of miR-30a is also of interest because the miR-30 family is well studied but has not previously been reported as being involved in vascular changes after SAH. The miR-30 family members are known for their role in angiogenesis-related myocardial matrix remodeling via their interaction with connective tissue growth factor [37].

Both miR-30a and miR-143 were upregulated in cerebral vessels after SAH; however, this regulation was not detectable in the serum. Furthermore, none of the other 10 investigated miRNAs showed significant changes in serum levels when compared to sham. Thus, none of the 12 candidate miRNAs show obvious potential as biomarkers for cerebral vasospasm and LCI after SAH. The cerebral vessels constitute a small amount of tissue compared to other cell types, and miRNAs secreted from the vessels after SAH might therefore be concealed by the miRNAs secreted from more abundant tissues. Another issue is the question of when or if the miRNAs can pass the blood–brain barrier and enter the blood stream. In general, miRNAs are small and might to some extent pass the blood–brain barrier, whose integrity is maintained by a delicate cross-talk between cerebral endothelial cells, junction proteins, and other cells of the neurovascular unit. In fact, recent data have suggested that miR-29b is involved in its regulation [38]. Even if regulated miRNAs from brain and/or cerebrovascular smooth muscle cells pass and in some cases possibly modify the blood–brain barrier, the potential for these miRNAs to serve as prognostic biomarkers seems, based on our findings, very limited for SAH because the major differences were seen early on (6 h post SAH) and normalized fairly rapidly to the sham level.

## Conclusion

The data from this miRNA screen in cerebral arteries of rats following SAH suggest that in particular, miR-30a and miR-143 changed with time but did not differ from a control group of sham-operated rats at 24 h. Future work with antagomirs will likely shed more light on the involvement of miRNAs in cerebral vasospasm, vascular inflammation, blood–brain barrier dysfunction, and development of LCI after SAH.

## Methods

### Experimental subarachnoid hemorrhage

All animal experiments were approved by the Danish Animal Experiments Inspectorate (license number: 2012-15-2934-00389).

Rats were subjected to experimental SAH following the model described by Prunell et al. [39]. Male Sprague–Dawley rats (Taconic, Denmark) weighing 320 g were anesthetized using 1.5% isoflurane (Baxter A/S, Denmark). Two catheters were placed, one in the tail artery to measure blood pressure and one in the subarachnoid space under the suboccipital membrane to measure the ICP. A laser Doppler probe was used to measure local cortical blood flow to ensure that the reduction in flow was of a sufficient magnitude and duration. A 27G canula was stereotactically inserted 6.5 mm anterior to the bregma at the midline and lowered until it hit the base of the skull, then retracted 1 mm. The rats were equilibrated for 15 min, and 250 μl blood was drawn from the tail catheter and injected via the canula at a pressure equal to the mean arterial blood pressure (80–100 mmHg). Only animals that displayed a drop in flow of 75% that remained low after a minimum of 5 min and a rise in ICP above mean arterial blood pressure were included in the study. Rats in the 0 h group were immediately drained of blood and euthanized by decapitation. The rats in the other groups were allowed to wake up and recover, and 7.5 mg/kg carprofen (Rimadyl®, Pfizer) was administered subcutaneously as analgesia. After 1 h, 6 h, or 24 h, the rats were anesthetized by subcutaneous injection with 2.5 ml/kg of a mixture of Hypnorm-Midazolam (1:1:2) in sterile water (containing 0.079 mg/ml fentanyl, 2.50 mg/ml fluanisone, Hypnorm®, VetaPharma Ltd, UK, and 1.25 mg/ml Midazolam-Hameln, Hameln, Germany). Rats were drained of blood by puncture of the ophthalmic venous plexus with a capillary tube, and serum was obtained. The rats were euthanized by decapitation, and the middle cerebral arteries (MCAs) and the basilar artery were dissected. Sham-operated rats underwent the same procedures as described above with the exception that no blood was injected intracisternally. Injection of saline was deliberately avoided because both the rise in ICP and the injected blood contribute to SAH-induced effects in the cerebral vasculature [20].

Rats were terminated at four time points (0 h, 1 h, 6 h, and 24 h) after SAH. Each time point contained four replicates, and each replicate consisted of tissue pooled from two animals. Animals subjected to sham operation were terminated at 24 h post-surgery and used as controls.

### RNA purification and qPCR

The MCAs and the basilar arteries of two animals were pooled (approx. 10 mg tissue in total), and total RNA was purified using a NucleoSpin miRNA kit (Macherey-Nagel, Germany) as described by the manufacturer. Tissue was pooled to extract a sufficient amount of RNA (40 ng). The RNA integrity number (RIN) was determined with an Agilent RNA 6000 Nano Kit on a 2100 Bioanalyzer (Agilent, USA) and a minimum of 7 was set as the inclusion criterion. Concentrations were determined with NanoDrop 2000c (Thermo Scientific, USA) spectrophotometer measurements. RNA was converted to cDNA using the Universal cDNA synthesis kit at 60 min at 42°C and 5 min at 95°C, and cooled to 4°C (Exiqon, Denmark). The qPCR screen of the miRNAome was performed with microRNA Ready-to-Use PCR Mouse & Rat Panel I + II V2 R with primers for 752 rodent miRNAs (728 of these are present in rat). This screen is a qPCR screen that includes two 384-well panels pre-coated with different primers in each well. All reagents including primers are based on the LNA™ technology patented by Exiqon, Denmark and commercially obtained from Exiqon, Denmark. The reactions were performed on a Bio-Rad CFX384 (10 min at 95°C with 45 amplification cycles at 95°C 10 s, 60°C 1 min, ramp-rate 1.6°C/s optical read), according to the manufacturer’s instructions (all reagents from Exiqon, Denmark).

To adjust for run-to-run variations, an interplate calibrator assay called UniSp3 in which both primers and DNA are present in the well was used. Only miRNAs present in all groups and in 60% of the samples within a group were included. Outlier detection was performed with Grubb’s test with a confidence level of 0.95 and cut-off SD of 0.25. Samples with Ct values over 37 were considered background and excluded from further analysis.

The qPCR data were analyzed using the 2^–ΔCt^ quantitative method [40], where ΔCt = Ct _target_ - Ct _global mean_. Normalization was performed to a global mean—i.e., the average of all microRNAs expressed in all samples with a Ct above 34 [41]—using the GenEX Pro 11 software (Multid analyses, Sweden). Data were analyzed with one-way ANOVA. Hierarchical clustering was performed with Ward’s algorithm and Euclidian distance measurements.

Technical confirmation of the candidate miRNAs from the miRNAome screen was carried out in triplicate using the same RNA template and primers as for the screen. The primers are based on Exiqon’s patented LNA™ technology and all primers were commercially obtained from Exiqon, Denmark. The reactions were performed in a 96-well plate-format (Pick-&-Mix panel from Exiqon) on a Roche Lightcycler 480 (10 min at 95°C, 45 amplification cycles at 95°C 10 s, 60°C 1 min, ramp-rate 1.6°C/s optical read), according to the manufacturer’s instructions. Samples with Ct values over 37 were considered background and excluded from further analysis. Expression was normalized to miR-103 as suggested by the “Norm-finder” algorithm, a model-based variance estimation predicting the stability of a given miRNA across samples [42]. The selection of miR-103 for normalization was made by subjecting the data from the technical confirmation to the “Norm-finder” algorithm. The qPCR data for the technical confirmation were analyzed using the 2^–ΔΔCt^ quantitative method [40] where ΔΔCt = (Ct _target_ - Ct _miR-103_)_SAH_ - (Ct _target_ - Ct _miR-103_)_Sham_ and statistically evaluated by one-way ANOVA followed by Student’s t-tests.

RNA from 50 μl serum was purified using the Serum NucleoSpin miRNA kit (Macherey-Nagel, Germany) as described by the manufacturer. RNA quality was assessed with an Agilent RNA 6000 Nano Kit on a 2100 Bioanalyzer (Agilent, USA) and RNA was converted to cDNA using the Universal cDNA synthesis kit at 60 min at 42°C and 5 min at 95°C, and cooled to 4°C. qPCR was performed using Pick-&-Mix panels with primers for 22 miRNAs. The reactions were performed on an ABI 7500 Fast (10 min at 95°C, 45 amplification cycles at 95°C 10 s, 60°C 1 min, ramp-rate 1.6°C/s optical read), according to the manufacturer’s instructions (all reagents from Exiqon, Denmark). Normalization was performed to miR-103a-3p, miR-107, miR-181a-3p, miR-181a-5p, miR24-3p, miR-451a, let-7i-5p, and miR23-3p. Differences between groups were tested with one-way ANOVA.

## Additional file

Additional file 1:
**482 miRNAs expressed in cerebral vessels of both sham and SAH rats at 1, 6, and 24 h post-operation.**
